# Reassortment Patterns in Swine Influenza Viruses

**DOI:** 10.1371/currents.RRN1008

**Published:** 2009-10-21

**Authors:** Hossein Khiabanian, Vladimir Trifonov, Raul Rabadan

**Affiliations:** Columbia University

## Abstract

Previous human influenza pandemics were the results of emerging viruses from non-human reservoirs, with at least two caused by strains of mixed human and avian origin. Also, many cases of swine influenza viruses have reportedly infected humans, including the recent human H1N1 strain, isolated in Mexico and the United States. Pigs are documented to get infected with human, avian, and swine viruses and allow productive replication, thus it has been conjectured that they are the “mixing vessel” that create reassortant strains, causing the human pandemics. In this paper, we apply several statistical techniques to an ensemble of publicly available swine viruses to study the reassortment phenomena. The reassortment patterns in swine viruses confirm previous results found in human viruses that the glycoprotein coding segments reassort most often. Moreover, one of the polymerase segments (PB1), reassorted in the strains responsible for the last two human pandemics of 1957 and 1968, also reassorts frequently.

## Introduction

Pandemics are epidemics that rapidly spread on a worldwide scale, caused by pathogens against which humans have no immunity that infect a large part of the population and lead to associated serious illnesses. Human influenza pandemics are caused by emerging influenza viruses from non-human reservoirs. From the three influenza pandemics of the twentieth century, the 1918 pandemic was possibly caused by an influenza virus with an avian origin [1] [2] and the other two, in 1957 and 1968, were caused by new strains that were combinations of avian and human viruses through the process of reassortment [3] [4]. 

There also have been many cases of swine influenza viruses infecting humans [5] [6]. In particular, in March 2009, a new human H1N1 influenza A virus of swine origin was isolated in Mexico and the United States [7] [8]. Preliminary analysis of the genome of this strain indicated that it is a descendant of common reassortant swine influenza A viruses [9]. Moreover, since 2003, a highly pathogenic H5N1 avian virus has been successfully infecting more than 400 humans with a mortality rate of 60% [10]. It is not clear whether any of these viruses will be the cause of the next human influenza pandemic, however, it is vital to understand the mechanisms behind the genomic evolution of influenza virus and its adaption to new hosts, in particular through the process of reassortment. 

Influenza A virus can be found in humans and a variety of animals with aquatic birds being considered as its main reservoir. Influenza viruses do not usually transmit between different hosts. However, pigs are documented to be infected with avian and human viruses, in addition to the swine viruses. Furthermore, multiple reassortment events are found to happen under natural conditions [11]. Hence, it has been postulated that swine are the mixing vessel for inter-host influenza viruses [12]. 

The influenza A virus genome consists of eight single-stranded RNA segments that code for eleven known proteins. The PB2, PB1, and PA segments encode the RNA polymerase, and HA, NP, NA, and M encode hemagglutinin, nucleoprotein, neuraminidase, and the matrix proteins, respectively. Two distinct non-structural proteins are also coded by the NS segment. The subtypes of influenza A viruses are determined based on their antigenic surface glycoproteins, hemagglutinin and neuraminidase. Hemagglutinin binds to α2,3-galactose- and α2,6-galactose-linked sialic acids. The former is more preferential in avian viruses and the later in human viruses. However they are both present on the tracheal epithelium surface in pigs, making them susceptible to both avian and human viruses. 

In addition to the genomic drift of influenza A virus that is caused by the high error rate in the process of replication of its genome, and the antigenic pressure on the HA and NA segments, the evolution of the virus is shaped by the reassortment process. When two different strains of influenza virus co-infect the same cell, new virions can be created that contain a mix of segments from both original strains. This phenomenon was responsible for the 1957 pandemic when the human H1N1 strain that had been circulating since 1918 reassorted to become a human H2N2 strain with new PB1, HA, and NA segments of avian origin [3] [4]. Also, in 1968, the reassortment of the PB1 and HA segments created a new human H3N2 strain which is currently co-circulating with the human H1N1 strain that reappeared in 1977 [4] [13] [14]. 

Swine classical H1N1 strains have been circulating in pigs since the human influenza pandemic in 1918 and were the dominant strains in the United States until 1998, when two new swine H3N2 strains were identified. These new strains were the results of a double reassortment of swine classical H1N1 with the PB1, HA, and NA segments from a human H3N2 strain, and a triple reassortment of swine classical H1N1, with the PB1, HA, and NA segments of a human H3N2 strain and the PB2 and PA segments of avian lineage [15] [16] [17]. So far, multiple strains of influenza virus (with various subtypes such as H1N2, H3N1, H2N3, H4N6, H5N1, etc.) have been isolated in pigs around the world, including both inter-host reassortments and whole genome adaptations of human and/or avian viruses [11] [18] [19] [20] [21] [22] [23] [24] [25] [26]. 

In this paper, we employ the temporally and geographically diverse information deposited in the Influenza Virus Resource of the National Center for Biotechnology Information [27] to study the reassortment phenomena in swine influenza A viruses. By integrating the information from the publicly available sequences, we investigate patterns in the reassortment events. Applying several statistical techniques, we identify the differential variability of the segments in the influenza genome and enumerate the independent reassortment events. These techniques include diversity/entropy measures of each segment and correlations between them. We confirm some of the previously reported results from human viruses that HA and NA reassort more frequently than the other segments [28] [29]. Surprisingly, we find that one of the polymerase segments, PB1, reassorts quite frequently, reiterating similar experimental results reported by Downie [30]. 

## Methods

To compare the diversity within the segments of swine influenza A virus, we use strains deposited in the Influenza Virus Resource of the NCBI that have all eight segments completely sequenced. We include 150 sequences, containing 99 H1N1, 25 H1N2, 23 H3N2, and 3 H3N1 strains (see Appendix). For each segment, we align the sequences of their coding regions using the Smith-Waterman algorithm and calculate the normalized Hamming distances only at the third codon positions, to eliminate the effects of evolutionary pressure due to positive selection. For the M and NS segments, we only consider the coding regions of the M1 and NS1 genes, as they are the longest and the most frequently sequenced sections of the M and NS segments. Because homologous recombination is very rare or absent in influenza viruses [31] [32], this restriction does not alter the results of our analysis. 

To measure the diversity of a segment i, we calculate D_i_, Rao’s quadratic entropy [33], according to

\begin{equation*}D_{i} = \frac{1}{N^2} \sum_{a, b}^{N} {d^{i}_{ab}}\end{equation*},

where N is the total number of strains in the dataset and diab is the Hamming distance between strains a and b at the third codon positions of their corresponding segment i. We estimate the confidence intervals for the diversity measurements via 1000 bootstrap re-samplings of the dataset. 

To find the possible reassortant strains, we primarily follow the method introduced by Rabadan et al. (2008), which was initially applied to complete sequences of human influenza A strains [29]. Briefly, in this method, the number of nucleotide differences between the segments of any two strains is calculated. Assuming that the segments have proportional substitution rates at the third codon positions, the differences between two segments of two strains should be proportional if the two segments have a common origin. A violation of this rule indicates that the histories of the two segments are different, i.e. there has been a reassortment event. Therefore, when the distances between two segments of different strains are plotted against each other, the points corresponding to possible reassortment events lie off the diagonal (Figure 1). 

**Figure:**
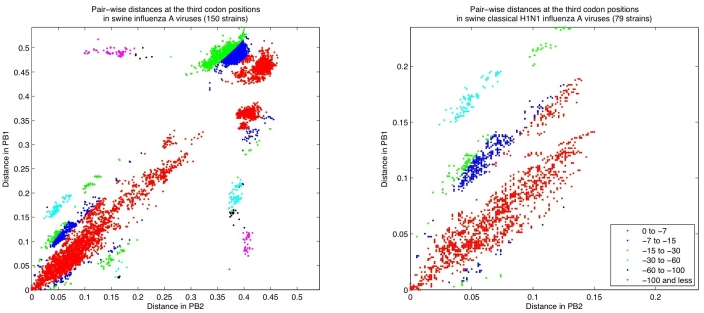
***Figure 1: Pair-wise hamming distances at the third codon positions in PB2 vs. PB1. The colors demonstrate the logarithm of the cumulative probability for the points, among which the ones with a cumulative probability of less than 10*** ^***-7***^ ***indicate possible reassortment events. Left: The results from 150 strains in the dataset, where there are candidates for reassortment events in both PB2 and PB1. Right: The results when the dataset is limited to the classical H1N1 strains isolated in the 70’s, 80’s, and 90’s, where there are distinctively more candidates for reassortment events in PB1.***

Given two strains a and b and two segments i and j, the probability to obtain hamming distances equal to diab and djab by random chance only is given by the hypergeometric distribution: 

\begin{equation*}P_{ab} (d^i_{ab}, L^i + L^j, d^i_{ab} + d^j_{ab}) = \frac{{L^i \choose d^i_{ab}}{L^j \choose d^j_{ab}}}{{L^i + L^j \choose d^i_{ab} + d^j_{ab}}}\end{equation*},

where L^i^ and L^j^ are the respective lengths of the segments divided by three. Hence, fixing the total distance between segments i and j of the two strains, the probability of observing a distance in segment i at most d^i^_ab_ is the cumulative of the hypergeometric distribution. Maintaining the assumption of similar average substitution rates at the third codon positions in all segments, in this model the lower the cumulative probability, the more likely it is that the two segments do not have a common ancestor. To correct for multiple hypotheses testing, for every two segments of each strain we generate 100 pairs of segments by randomly permuting their third codon positions. We observe that the cumulative probabilities for distances of pairs from the generated data are at least 10^-7^. Thus, a cumulative probability of at most 10^-7^ for two given segments of two strains indicates a reassortment event. 

Finally, for each of the 150 strains, we generate a list of strains with which they have low probabilities of having common ancestors, hinting to reassortment events. For further investigation of the origin of the segments, we compile a large target database of more than 10,800 strains of influenza A virus that includes all completely sequenced human and avian isolates, in addition to all swine isolates deposited in the Influenza Virus Resource of the NCBI. We use this database to compare the histories of two segments of a given swine strain. First, we align with NCBI BLAST [34] the two segments to the sequences in the target database, which precede in time the strain of interest. Second, we define the history overlap of the two segments as a function of the alignment identity in the following way. For a given alignment identity x, let Ix be the set of target strains with which the first segment has identity at least x. Similarly define Jx for the second segment. Then the history overlap for alignment identity x is the number of strains common to Ix and Jx over the number of strains included in either one of them. In general, low values of the history overlap function indicate distinct histories of the segments and high values correspond to common history. A decrease in the values of the history overlap function could indicate a potential fork in the lineage of one of the segments. Conversely, an increase can be the result of a merge of the lineages of the two segments, i.e. a reassortment event. Those observations allow us to confirm in an alternative and independent manner the reassortment events predicted by the hypergeometric probability analysis. The history overlap analysis is limited by the sequences present in the target database, but when enough data is available and the converging/diverging lineages are sufficiently different, it can provide a good indicator of the corresponding event. 

For a demonstration of the analyses described above consider the strain A/swine/Tennesse/23/1976. When compared to A/swine/Iowa/1/1976 the hamming distance in the PB1 segments is 11% and the hamming distance in the NP segments is 3%. The cumulative hypergeometric probability of this event is less than 10-7, which indicates a reassortment event in at least one of those strains at either segment PB1 or NP. The history overlaps for those two segments and the rest of the segments of A/swine/Tennesse/23/1976 are shown in Figure 2. The figure shows that NP and all the other segments except PB1 share a common recent history, whereas the recent history of PB1 is different from the other seven segments. This allows us to assert that the PB1 segment of A/swine/Tennesse/23/1976 is the result of a reassortment. An interesting feature apparent in Figure 2 is that at lower identities M1 shares fewer strains with PB1 and NP. This observation can be attributed to a possible slower evolutionary rate of M1 and a fork in its lineage to a line of human viruses. Similar considerations show that the PB1 segment of A/swine/Iowa/1/1976 is also the result of a reassortment, however the PB1 segments of A/swine/Iowa/1/1976 and A/swine/Tennessee/23/1976 are from different lineages and the target database contains isolates close to the former, but not the latter.

**Figure:**
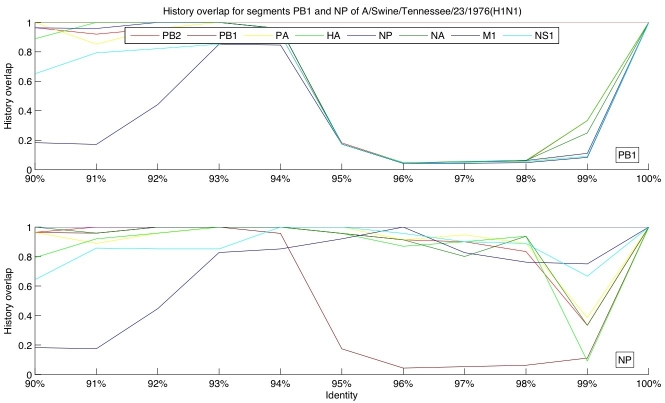
***Figure 2: History overlap for segments PB1 and NP of the swine influenza strain A/Swine/Tennessee/23/1976. NP and all the other segments except PB1 share a common recent history, whereas the recent history of PB1 is different from the other seven segments, indicating a reassortment event at PB1. The small history overlap of M1 with PB1 and NP at lower identities can be attributed to a possible slower evolutionary rate of M1 and a fork in its lineage to a line of human viruses. The fluctuations in the history overlap of NP at 99% identity are due to small number of sample points at that level of identity.***

## Results and Discussion

Viruses present an enormous diversity due to their high mutation rate, short replication time, and high number of replicates. There are several ways of measuring the diversity of a viral population: richness, evenness, Rao’s entropy [33], Shannon entropy, other Renyi entropies, etc. When applied to actual viral populations, all these measures encounter similar problems: sampling bias (for instance, most of human influenza samples come from a few studies in New York State and New Zealand [27]), exponential growth and bottleneck structures of viral populations, population stratification, etc. Although the exact interpretation of these measures applied to highly structured populations is not clear, they can be used to compare the variation of diversity in different sections of the genome of a particular organism. Since similar histories imply similar diversity measures, strong differences in these measures for different sections of the genome point to their different histories. 

Although the third codon evolutionary rates in influenza A viruses are thought to be similar in all segments, the analysis of the genomic diversity of the strains in our dataset reveals a very inhomogeneous pattern. Figure 3 (left), shows Rao’s quadratic entropy, measured at the third codon positions, for 150 swine influenza A viruses that have all 8 fully sequenced segments deposited in NCBI, along with the 95% bootstrap percentile confidence intervals. This figure indicates a statistically significant difference between NA, HA, and PB1 compared to PB2, PA, NP, M, and NS. Moreover, within a particular subtype, where the variations in HA and NA are fixed, PB1 appears as the most diverse segment. Figure 3 (right) shows the diversity in the swine classical H1N1 strains that were isolated in the 70’s, 80’s and 90’s. This analysis shows that the eight segments do not have a common history, with PB1, HA, and NA presenting a higher level of diversity. 

**Figure:**
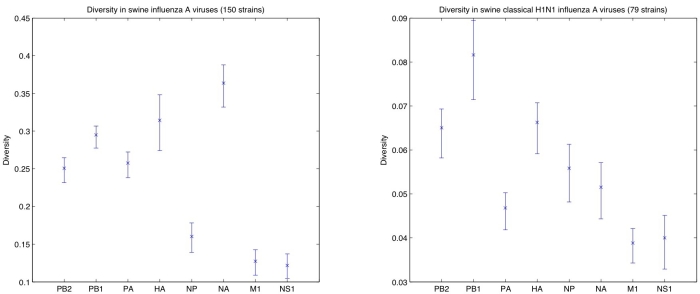
***F*** ***igure 3: Diversity measurements in swine influenza viruses and the corresponding 95% bootstrap percentile confidence intervals. Left: Considering the 150 strains in the dataset, NA, HA, and PB1 present a higher diversity than the rest. Right: When the dataset is limited to the classical H1N1 strains isolated in the 70’s, 80’s, and 90’s, which fixes the HA and NA variations in the population, shows a higher diversity in PB1 than the rest of the segments.***

We further investigate the sources of variation in diversity via the pair-wise Pearson correlation of the distances at the third codon positions of the viral segments. Correlations, linear or non-linear, or any other measure of dependence, such as mutual information, encounter the same problems as those of the measures of diversity (sampling bias, bottleneck structures, population stratification, etc.). Nonetheless, they are revealing indicators of the origins of diversity in a population. When all the 150 strains in the dataset are considered, the correlations are lower between the surface glycoprotein coding segments and the other segments. More interestingly, the PB1 segment also has a low correlation with all segments that are not polymerase coding (Figure 4, left). Especially, when the strains from a particular subtype are considered and the variations in HA and NA segments are fixed in the dataset, PB1 presents the least correlation relative to the other segments. This is evident, among the classical swine H1N1 strains isolated in the 70’s, 80’s and 90’s (Figure 4, right).

**Figure:**
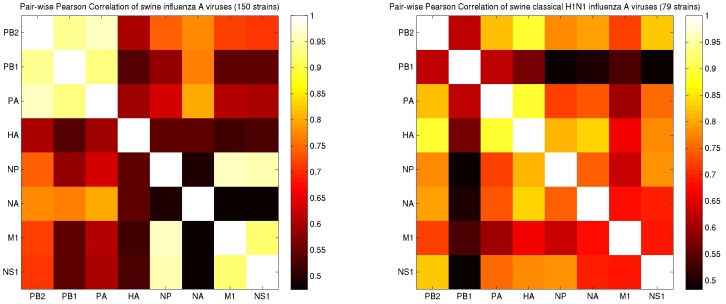
***Figure 4: Pair-wise Pearson correlation of the distances at the third codon positions of the viral segments. Left: The HA, NA, and PB1 segments have the least correlation in regards to the rest of the segments. Right: When the HA and NA variations are fixed in the population by limiting the dataset to the classical H1N1 strains isolated in the 70’s, 80’s, and 90’s, PB1 presents a distinctively lower correlation relative to the other segments.***

The above observations from the diversity and pair-wise correlation measures hint to distinct evolutionary patterns in the HA, NA, and PB1 segments. To elucidate the role of the process of reassortment in these patterns, we have enumerated the independent reassortment events in swine viruses that we identify through the hypergeometric distribution analysis of Rabadan et al. (2008) [29] and confirm via history overlap analysis, described in the Methods section. Table 1 lists these events, represented by different strains and a simple inspection reveals the frequent role of NA, HA, and PB1 in the reassortment process. Because the reassortment events are frequent in swine viruses and the sampling is not, it is difficult to determine their exact history. However, especially in cases where there are multiple reassortments, we have attempted to identify the fully sequenced strains that are the earliest independent isolations of the reassortant viruses. As indicated in the last column of Table 1, some of the listed strains have been already published independently. In addition, for a more comprehensive list, we have included the reported reassortment events for which there are no completely sequenced isolates available, so that they could not be identified by our method. Finally, we have listed other published reassortant strains, which according to Krasnitz et al. (2008) either are “frozen in time” or show evidence of homologous recombination [31]. 

To summarize, our analyses show that not every segment of the swine influenza virus reassorts in equal fashion. In accordance with the previous results from human influenza A viruses, both in vitro [28] and in vivo [29], we find that the surface glycoproteins coding segments (HA and NA) reassort at a higher rate. Perhaps, the most intriguing conclusion of our analyses is the characteristic role of one of the polymerase coding segments (PB1) that appears frequently in both inter-host and intra-host reassortment events. Interestingly, this is the same pattern observed in the strains responsible for the 1957 and 1968 pandemics, when human viruses also obtained PB1 segments of avian origin. 

The mechanisms behind the preferential reassortments are not clear, however several hypotheses can be advanced. There is substantial evidence for biases in the packaging mechanism of the viral RNA into the virion for influenza A viruses, which can impose a selective pressure on segments that can be exchanged between strains [35]. Another constraint on the reassortment events can be associated with compensatory mutations due to interactions between the different proteins.

## Funding Information

The authors have no support or funding to report.

## Competing Interests

The authors have declared that no competing interests exist.

## Tables

**Table 1:** Summery of the reassortment events in swine influenza A viruses. (The notation is S: swine, A: avian, and H: human and the numbers indicate the different origins of the segments and distinguish different host lineages.)

**Table:** 

**Year**	**Strain**	**Subtype**	**PB2**	**PB1**	**PA**	**HA**	**NP**	**NA**	**MP**	**NS**	**Ref.**
1976	A/swine/Iowa/1/1976	H1N1	S1	S2	S1	S1	S1	S1	S1	S1	
1976	A/swine/Tennessee/15/1976	H1N1	S1	S2	S1	S1	S1	S1	S1	S1	
1976	A/swine/Tennessee/19/1976	H1N1	S1	S1	S1	S1	S1	S1	S2	S1	
1976	A/swine/Tennessee/23/1976	H1N1	S1	S2	S1	S1	S1	S1	S1	S1	
1977	A/swine/Tennessee/48/1977	H1N1	S1	S1	S2	S1	S1	S1	S2	S1	
1977	A/swine/Tennessee/61/1977	H1N1	S1	S1	S1	S1	S1	S1	S2	S1	
1977	A/swine/Tennessee/62/1977	H1N1	S1	S1	S1	S1	S1	S2	S2	S1	
1977	A/swine/Tennessee/64/1977	H1N1	S1	S2	S1	S1	S1	S1	S1	S1	
1977	A/swine/Tennessee/82/1977	H1N1	S1	S1	S1	S2	S1	S2	S1	S1	
1977	A/swine/Tennessee/96/1977	H1N1	S1	S1	S1	S1	S1	S1	S1	S2	
1979	A/swine/Minnesota/5892-7/1979	H1N1	S1	S1	S1	S1	S2	S1	S1	S1	
1981	A/swine/Ontario/6/1981	H1N1	S1	S1	S1	S1	S2	S1	S1	S1	
1986	A/swine/Iowa/1/1986	H1N1	S1	S1	S2	S2	S1	S1	S1	S1	
1988	A/swine/Wisconsin/1915/1988	H1N1	S1	S1	S1	S1	S2	S1	S1	S1	
2004	A/swine/Korea/CAN01/2004	H1N1	S1	S1	S1	S1	S1	S2	S1	S1	[25]
2004	A/swine/Spain/53207/2004	H1N1	S1	S1	S1	S2	S1	S2	S1	S3	
2007	A/swine/Ohio/24366/07	H1N1	S1	S1	S1	S2	S1	S2	S1	S1	
											
1998	A/swine/Italy/1521/98	H1N2	S1	S1	S1	S2	S1	S3	S1	S1	[18]
1999	A/Swine/Indiana/9K035/99	H1N2	S1	S1	S1	S2	S1	S1	S1	S1	[36]
2000	A/Swine/Minnesota/55551/00	H1N2	S1	S1	S1	S2	S1	S1	S1	S1	[37]
2004	A/swine/Zhejiang/1/2004	H1N2	S1	S1	S1	S1	S1	H	S1	S1	[19]
2005	A/swine/Cloppenburg/IDT4777/2005	H1N2	S1	S1	S1	S1	S1	S2	S1	S1	[20]
2006	A/swine/Miyazaki/1/2006	H1N2	S1	S1	S1	S1	S1	S3	S1	S1	[21]
2007	A/swine/Shanghai/1/2007	H1N2	S1	S1	S1	S2	S1	S1	S1	S1	
											
1998	A/Swine/Nebraska/209/98	H3N2	A	H	A	H	S	H	S	S	[36]
2001	A/swine/Spain/33601/2001	H3N2	S1	S1	S1	S2	S1	S2	S1	S1	
2003	A/swine/North Carolina/2003	H3N2	S	S	S	H1	S	H2	S	S	
2007	A/swine/Korea/CY04/2007	H3N2	S1	S1	S1	S1	S1	S1	S1	S2	[25]
2007	A/swine/Korea/CY07/2007	H3N2	S1	S1	S2	S2	S1	S1	S1	S1	[25]
											
	**Other published (incompletely sequenced) strains **										
1998	A/swine/North Carolina/35922/98	H3N2	S	H	S	H	S	H	S	S	[16]
2004	A/swine/MI/PU243/04	H3N1	S1	S1	S1	S1	S1	S2	S1	S1	[38]
2006	A/swine/Missouri/2124514/2006	H2N3	S1	S2	A	A	S1	A	S1	S1	[39]
											
	**Strains “frozen in time” or with evidence of homologous recombination [31]**										
2003	A/swine/Alberta/56626/03	H1N1	S1	S1	S2	S1	S1	S3	S1	S1	[40]
2003	A/swine/Ontario/53518/03	H1N1	S3	S3	S2	S1	S3	S1	S1	S1	[40]
2003	A/swine/Ontario/57561/03	H1N1	S1	S1	S2	S1	S3	S1	S2	S1	[40]
2004	A/swine/Ontario/48235/04	H1N2	S1	H1	S1	H2	S2	H3	S3	S3	[40]
2004	A/swine/Ontario/11112/04	H1N1	S1	H	S1	S1	S2	S1	S1	S1	[40]
2005	A/swine/Alberta/14722/2005	H3N2	S	S	S	S	S	H	S	S	[41]

## References

[ref-3707357317] Taubenberger JK, Reid AH, Lourens RM, Wang R, Jin G, et al. (2005) Characterization of the 1918 influenza virus polymerase genes. Nature 437(7060): 889-93. 10.1038/nature04230 16208372

[ref-2463909567] Rabadan R, Levine AJ, Robins H (2006) Comparison of avian and human influenza A viruses reveals a mutational bias on the viral genomes. J Virol 80(23): 11887-91. 10.1128/JVI.01414-06 PMC1642607 16987977

[ref-3148427921] Lindstrom SE, Cox NJ, Klimov A (2004) Genetic analysis of human H2N2 and early H3N2 influenza viruses, 1957-1972: evidence for genetic divergence and multiple reassortment events. Virology 328(1): 101-19. 10.1016/j.virol.2004.06.009 15380362

[ref-1631022973] Scholtissek C, Rohde W, Von Hoyningen V, Rott R (1978) On the origin of the human influenza virus subtypes H2N2 and H3N2. Virology 87(1): 13-20 10.1016/0042-6822(78)90153-8 664248

[ref-2121853709] Myers KP, Olsen CW, Gray GC (2007) Cases of Swine Influenza in Humans: A Review of the Literature. Clin Infect Dis 44(8): 1084–1088. 10.1086/512813 PMC1973337 17366454

[ref-881861887] Shinde V, Bridges CB, Uyeki TM, Shu B, Balish A, et al. (2009) N Engl J Med 360(25): 2616-25. 10.1056/NEJMoa0903812 19423871

[ref-3815587315] Center for Disease Control (2009) Swine Influenza A (H1N1) Infection in Two Children - Southern California, March - April 2009. MMWR 58(15); 400-402. 19390508

[ref-2891251973] Center for Disease Control (2009) Outbreak of Swine-Origin Influenza A (H1N1) Virus Infection - Mexico, March - April 2009. MMWR 58(Dispatch): 1-3. 19444150

[ref-11962701] Trifonov V, Khiabanian H, Greenbaum B, Rabadan R (2009) The origin of the recent swine influenza A(H1N1) virus infecting humans. Euro Surveill 14(17): pii=19193. 19422769

[ref-3488636989] Komar N, Olsen B (2008) Avian Influenza Virus (H5N1) Mortality Surveillance. Emerg Infect Dis 14(7): 1176-1178. 10.3201/eid1407.080161 PMC2600356 18598659

[ref-2923360535] Brown IA (2000) The epidemiology and evolution of influenza viruses in pigs. Vet Microbiol 74(1-2): 29-46. 10.1016/s0378-1135(00)00164-4 10799776

[ref-2789652485] Scholtissek C (1990) Pigs as the ‘mixing vessel’ for the creation of new pandemic influenza A viruses. Med Princip Prac 2: 65-71.

[ref-1826350897] Nakajima K, Desselberger U, Palese P (1978) Recent human influenza A (H1N1) viruses are closely related genetically to strains isolated in 1950. Nature 274(5669): 334-339. 10.1038/274334a0 672956

[ref-3643259761] Scholtissek C, von Hoyningen V, Rott R (1978) Genetic relatedness between the new 1977 epidemic strains (H1N1) of influenza and human influenza strains isolated between 1947 and 1957 (H1N1). Virology 89(2): 613-617. 10.1016/0042-6822(78)90203-9 716220

[ref-3213076669] Webby RJ, Swenson SL, Krauss SL, Gerrish PJ, Goyal SM, et al. (2000) Evolution of swine H3N2 influenza viruses in the United States. J Virol 74(18): 8243-51. 10.1128/jvi.74.18.8243-8251.2000 PMC116332 10954521

[ref-1555805227] Zhou NN, Senne DA, Landgraf JS, Swenson SL, Erickson G, et al. (1999) Genetic reassortment of avian, swine, and human influenza A viruses in American pigs. J Virol 73(10): 8851-6. 10.1128/jvi.73.10.8851-8856.1999 PMC112910 10482643

[ref-657581123] Vincent AL, Ma W, Lager KM, Janke BH, Richt JA (2008) Swine influenza viruses: a North American perspective. Adv Virus Res 72: 127-54. 10.1016/S0065-3527(08)00403-X 19081490

[ref-1015103605] Marozin S, Gregory V, Cameron K, Bennett M, Valette M, et al. (2002) Antigenic and genetic diversity among swine influenza A H1N1 and H1N2 viruses in Europe. J Gen Virol 83(Pt 4): 735-745. 10.1099/0022-1317-83-4-735 11907321

[ref-1236214273] Qi X, Lu CP (2006) Genetic characterization of novel reassortant H1N2 influenza A viruses isolated from pigs in southeastern. Arch Virol 151(11): 2289-99. 10.1007/s00705-006-0796-x PMC7087176 16755371

[ref-2296502039] Zell R, Motzke S, Krumbholz A, Wutzler P, Herwig V, et al. (2008) Novel reassortant of swine influenza H1N2 virus in Germany. J Gen Virol 89(PT 1): 271-276. 10.1099/vir.0.83338-0 18089751

[ref-2733682395] Saito T, Suzuki H, Maeda K, Inai K, Takemae N, et al. (2006) Molecular characterization of an H1N2 swine influenza virus isolated in Miyazaki, Japan, in 2006. J Vet Med Sci 70(4): 423-7. 10.1292/jvms.70.423 18460842

[ref-1734885653] Ludwig S, Stitz L, Planz O, Van H, Fitch WM, Scholtissek C. (1995) European swine virus as a possible source for the next influenza pandemic? Virology 212(2): 555-61. 10.1006/viro.1995.1513 7571425

[ref-3985195105] Castrucci MR, Donatelli I, Sidoli L, Barigazzi G, Kawaoka Y, Webster RG (1993) Genetic reassortment between avian and human influenza A viruses in Italian pigs. Virology 193(1): 503-6. 10.1006/viro.1993.1155 8438586

[ref-3454163905] Chutinimitkul S, Thippamom N, Damrongwatanapokin S, Payungporn S, Thanawongnuwech R, Amonsin A, et al. (2008) Genetic characterization of H1N1, H1N2 and H3N2 swine influenza virus in Thailand. Arch Virol 153(6): 1049-56. 10.1007/s00705-008-0097-7 18458812

[ref-3354822437] Yoo GJ, Kim CJ, Choi YK (2008) Seroprevalence and genetic evolutions of swine influenza viruses under vaccination pressure in Korean swine herds. Virus Res 138 (1-2): 43-49. 10.1016/j.virusres.2008.08.005 18789984

[ref-170079833] Karasin AI, Brown IH, Carman S, Olsen CW (2000) Isolation and characterization of H4N6 avian influenza viruses from pigs with pneumonia in Canada. J Virol 74(19): 9322-7. 10.1128/jvi.74.19.9322-9327.2000 PMC102133 10982381

[ref-2378519527] Bao Y, Bolotov P, Dernovoy D, Kiryutin B, Zaslavsky L, et al. (2008) The Influenza Virus Resource at the National Center for Biotechnology Information. J Virol 82(2): 596-601. 10.1128/JVI.02005-07 PMC2224563 17942553

[ref-2517101837] Lubeck MD, Palese P, Schulman JL (1979) Nonrandom association of parental genes in influenza A virus recombinants. Virology 95(1): 269-74. 10.1016/0042-6822(79)90430-6 442543

[ref-3797712659] Rabadan R, Levin AJ, Krasnitz M (2008) Non-random reassortment in human influenza A viruses. Influenza and Other Respiratory Viruses 2(1): 9-22. 10.1111/j.1750-2659.2007.00030.x PMC4634327 19453489

[ref-647720485] Downie JC (2004) Reassortment of influenza A virus genes linked to PB1 polymerase gene. International Congress Series 1263: 714-718.

[ref-2791085205] Krasnitz M, Levine AJ, Rabadan R (2008) Anomalies in the Influenza Virus Genome Database: New Biology or Laboratory Errors? J Virol 82(17): 8947-8950. 10.1128/JVI.00101-08 PMC2519662 18579605

[ref-136272173] Boni MF, Zhou Y, Taubenberger JK, Holmes EC (2008) Homologous Recombination Is Very Rare or Absent in Human Influenza A Virus. J Virol 82(10): 4807-4811. 10.1128/JVI.02683-07 PMC2346757 18353939

[ref-1108860715] Rao CR (1982) Diversity and dissimilarity coefficients: a unified approach. Theor Popul Biol 21(1): 24-43.

[ref-758965145] Altschul SF, Madden TL, Schaffer AA, Zhang J, Zhang Z, et al. (1997) Gapped BLAST and PSI-BLAST: a new generation of protein database search programs. Nucleic Acids Res 25:3389-3402. 10.1093/nar/25.17.3389 PMC146917 9254694

[ref-4169312935] Marsh GA, Rabadan R, Levine AJ, Palese P (2008) Highly conserved regions of influenza a virus polymerase gene segments are critical for efficient viral RNA packaging. J Virol 82(5): 2295-304. 10.1128/JVI.02267-07 PMC2258914 18094182

[ref-1251113741] Karasin AI, Schutten MM, Cooper LA, Smith CB, Subbarao K, et al. (2000) Genetic characterization of H3N2 influenza viruses isolated from pigs in North America, 1977-1999: evidence for wholly human and reassortant virus genotypes. Virus Res 68(1): 71-85. 10.1016/s0168-1702(00)00154-4 10930664

[ref-3603757663] Karasin AI, Landgraf J, Swenson S, Erickson G, Goyal S, et al. (2002) Genetic characterization of H1N2 influenza A viruses isolated from pigs throughout the United States. J Clin Microbiol 40(3): 1073-1079. 10.1128/JCM.40.3.1073-1079.2002 PMC120269 11880444

[ref-726278859] Lekcharoensuk P, Lager KM, Vemulapalli R, Woodruff M, Vincent AL, et al. (2006) Novel swine influenza virus subtype H3N1, United States. Emerg Infect Dis 12(5): 787-94. 10.3201/eid1205.051060 PMC3374457 16704839

[ref-271750471] Ma W, Vincent AL, Gramer MR, Brockwell CB, Lager KM, et al. (2007) Identification of H2N3 influenza A viruses from swine in the United States. Proc Natl Acad Sci U S A 104(52): 20949-20954. 10.1073/pnas.0710286104 PMC2409247 18093945

[ref-1759949381] Karasin AI, Carman S, Olsen CW (2006) Identification of human H1N2 and human-swine reassortant H1N2 and H1N1 influenza A viruses among pigs in Ontario, Canada (2003 to 2005). J Clin Microbiol 44(3): 1123-6. 10.1128/JCM.44.3.1123-1126.2006 PMC1393092 16517910

[ref-29509495] Olsen CW, Karasin AI, Carman S., Li Y, Bastien N, et al. (2006) Triple reassortant H3N2 influenza A viruses, Canada. Emerg Infect Dis 12 (7): 1132-1135. 10.3201/eid1207.060268 PMC3291069 16836834

